# Retraction: “A Case-Cohort Study of Cadmium Body Burden and Gestational Diabetes Mellitus in American Women”

**DOI:** 10.1289/EHP1818

**Published:** 2017-03-31

**Authors:** Megan E. Romano, Daniel A. Enquobahrie, Christopher D. Simpson, Harvey Checkoway, Michelle A. Williams

Environ Health Perspect 123(10):993–998 (2015), http://dx.doi.org/10.1289/ehp.1408282

This article is being retracted at the request of the authors because of inadvertent errors in the statistical code that resulted in the exclusion of 12 gestational diabetes mellitus cases with urinary cadmium below the limit of detection. The coding errors do not impact any other published studies of this population.

In Table 1, the corrected geometric mean for the gestational diabetes mellitus cases was 0.33 μg/g Cr [95% confidence interval (CI): 0.30, 0.37].

In Table 2, the magnitude of the effect estimates reported was attenuated and the *p*-trend was no longer statistically significant, such that the odds ratios for gestational diabetes mellitus with increasing urinary cadmium tertile were 1.12 (95% CI: 0.64, 1.98) for middle versus low tertile and 1.34 (95% CI: 0.78, 2.29) for high versus low tertile; *p*-trend = 0.28.

The magnitude of the effect estimates reported in Table 3 and the effect estimates for additional sensitivity analyses reported in the original manuscript were also generally attenuated.

The authors regret any inconvenience to the scientific community.

